# Supplementary material for: Angiotensin-(3–4) normalizes the elevated arterial blood pressure and abnormal Na+/energy handling associated with chronic undernutrition by counteracting the effects mediated by type 1 angiotensin II receptors
Source: PLoS One. 2022 Aug 19;17(8):e0273385. doi: 10.1371/journal.pone.0273385 (PMC9390919; doi:10.1371/journal.pone.0273385)
Supplement: Attachment — Submitted filename: PONE-D-22-09686 - Response to Reviewers June 28th .docx [file pone.0273385.s001.docx]

**PONE-D-22-09686**

**Pereira-Acacio *et al.***

**Response to Reviewers**

The original manuscript (OM) entitled “Angiotensin-(3–4) normalizes the elevated arterial blood pressure and abnormal Na^+^/energy handling associated with chronic undernutrition by counteracting the effects mediated by type 1 angiotensin II receptors” has been extensively and thoroughly revised following the Reviewers’ recommendations. In the revised manuscript (RM), we addressed their comments point-by-point, as described below.

The modifications in the text are highlighted in yellow in the marked version of RM, “Revised Manuscript with Track Changes”, as requested. An unmarked version of your revised paper without tracked changes “Manuscript” is also uploaded, as requested.

The Reviewers’ suggestions were especially useful in improving the presentation and interpretation of the data, and we would like to express our appreciation for their careful and appropriate analysis.

**Academic Editor’s comments.**

**Comment.**

**Citation.** “*The both reviewers have concerned statistical analysis. Thus, the authors should address all of these comments satisfactory before resubmission.*”

**Answer.** We addressed the entire Reviewers’ comments, as the Academic Editor can see below.

**Reviewer 1.**

**Comment 1.**

**Citation. “***P values are presented as four decimal places, which is meaningless. This is animal study, therefore P<0.01, P<0.001 or two decimal places is enough. See the article in PloS One reported from your laboratory (PLoS One. 2014 Jul 1;9(7):e100410).*”

**Answer.** We accepted this recommendation. We now use p > 0.05, p < 0.05, p < 0.01 and p < 0.001 for “p” values throughout the RM. Please, see the modified figures and their corresponding legends. Since this recommendation does not apply to Figure 6, its corresponding legend remains unmodified in the RM.

**Comment 2.**

**Citation.** “*Presentation of Figures are not appropriate. In Fig 1, for example, the same values of CTR (-/-)and RBD (-/-) columns show 4 columns in the same figure.*”

**Answer.** We believe that the Reviewer refers to Figure 2 (plasma albumin concentration) in his(her) example, which is applicable to other figures. This criticism is addressed in several parts of the RM. We especially acknowledge this criticism that helped us to improve the statistical analysis and the presentation of the results as a whole.

1. In the RM we used the Student’s *t*-test only for the comparison of the 2 rate constants of growth *k* obtained with same function for CTRL and RBD rats in the absence of Losartan (page 9, lines 255 to 256; RM). In the presence of Losartan, we found 2 different time-dependencies and, therefore, the corresponding rate constants *k* cannot be compared with those obtained in the absence of Losartan. Please, see page 9 from line 249 to page 10, line 280; and also, legend to Figure 1 on page 27; RM.
2. We did not use the Student’s *t*-test to compare two groups within several groups (see below comment 3 by this Reviewer) throughout the RM. We used one-way ANOVA followed by Bonferroni’s test for the comparisons, as requested.
3. We removed the isolated comparison between the CTRL and the RBD groups (panels A in several figures of the OM) because the comparison is also possible in the other panels. The untreated CTRL and RBD rats are now compared in the new panel A of several figures in the RM; please see below points “4 and 5” in this response.
4. We maintained 3 panels in Figures 2, 7, 11, 13, and 14 (***i***) to compare the corresponding parameter between the CTRL and RBD rats and to analyze the effects of Losartan alone (new panel A), (***ii***) to analyze the effects of Ang-(3–4) alone (new panel B), and (***iii***) to analyze the effects of the combined treatment in both untreated CTRL and RBD rats (new panel C). Appropriate titles in the figure legends now clearly identify the aim of each panel. The entire legends have been rewritten, as well as the description of the figures in the Results section from the RM, as follows. Page 10, lines 285 to 290; page 13, lines 365 to 369; page 14, lines 390 to 401; page 15, lines 415 to 422.
5. In the case of Figures 3, 4, 5, 8 (supplementary Figure 1 in the OM), 9 (supplementary Figure 2 in the OM), 10 (Figure 8 in the OM), and Figure 12 (Figure 10 in the OM), the number of panels is four. This has been preserved (***i***) to compare the corresponding parameter between the CTRL and RBD rats and to analyze the effects of Losartan (new panel A), (***ii***) to analyze the effects of Ang-(3–4) alone (new panel B), (***iii***) to analyze the effects of Ang-(3–4) in rats that were chronically treated with Losartan (new panel C), and (***iv***) to analyze the effects of the combined treatment in both untreated CTRL and RBD rats (new panel D). Again, appropriate titles in the figure legends now clearly identify the aim of each panel, and the entire legends have been also rewritten, as well as the description of the figures in the Results section from the RM, as follows. Page 10, line 294 to page 12, line 316; page 12, lines 317 to 326; page 12, lines 333 to 344; page 13, lines 370 to 378; page 13, line 379 to page 14, line 383; page 14, lines 384 to 388; page 14, lines 402 to 408.

The aim of the presentation of these different panels is also mentioned on page 5, lines 152 to 153 (RM). Please, note that the lettering of the panels has changed and this is reflected in the presentation of the results.

6. Except for the insertions required by Reviewer 2, the content of the Discussion section remains unmodified in the RM, because the results and the ideas of the article did not change.

**Comment 3.**

**Citation.** “*Also, the authors misunderstand ANOVA. ANOVA is applied to estimate the strictly correct variance of the parameter of interest to avoid screwed variances. Therefore, all groups in Fig1 must be put in the same ANOVA for calculation. The authors cannot use Student t-test for analysis to compare two groups of several groups.*”

**Answer.** As pointed out above, we did not use the Student’s *t*-test to compare two groups within several groups in the RM. We used one-way ANOVA followed by Bonferroni’s test for the comparisons of body mass. With respect to Figure 1 (evolution of body mass with time of rats that received or not Losartan) we put the 4 groups in the same ANOVA within a single panel and, for this reason, we now write “Fig 1” and not “Fig 1A” or “Fig 1B” (see page 9 from line 249 to page 10, line 280. The differences were assessed weekly, and they are mentioned in the modified legend (page 27, lines 798 to 802; RM). Only the 2 rate constants of growth *k* of untreated CRTL and RBD rats, which were obtained with the same function, were compared using the Student’s *t*-test, as mentioned above in our response to comment 2 by this Reviewer.

**Comment 4.**

**Citation.** “*Since there are no restrictions on the number of figures, it is better not to use supplementary figures.*”

**Answer.** The supplementary Figures 1 and 2 in the OM are Figures 8 and 9 in the RM. Consequently, the numbering of Figures 8–12 changed to Figures 10– 14.

**Comment 5.**

**Citation.** “*Did the authors check the yield or purity of plasma membrane-rich fraction prepared for each ATPase activity from kidney proximal tubule cells among groups.*”

**Answer.** Two sentences regarding the preparation method, the control for residual contamination with membranes of intracellular organelles and the yield, addresses this point raised by the Reviewer (page 7, lines 186 to 198; page 7, line 203 to page 8, line 206; RM).

**Comment 6.**

**Citation.** “*Manuscript text should be double-spaced according to instruction for authors.*”

**Answer.** The RM is double-spaced throughout, as required.

**Reviewer 2.**

**Comment 1.**

**Citation.** “*Do the changes caused by RBD in the Wistar rats resemble that in human being, including Body weight, energy intake, Na+ ingestion, Na+ excretion, hypertension extent ?*”

**Answer.** Teodósio and coworkers [ref. 5] formulated this diet according to the dietary habits of populations from vast sugarcane regions in Northeast Brazil. The change caused by RBD in body weight resembles that encountered in humans. The studies on Na^+^ ingestion (together with energy intake), Na^+^ excretion, Na^+^ balance, and their relation with hypertension are original observations from the present study. A new sentence on page 3, lines 76 to 79 (RM), clearly states this similarity.

**Comment 2.**

**Citation.** “*Figure 7, the elevated SBP is almost normalized by Losartan in RBD group. Ang-(3-4) also has an hypotensive effect in RBD. Why the SBP seemed to increase SBP in the combination therapy group (Lorsartan+Ang-(3-4)) as compared to the single therapy group (Lorsartan or Ang-(3-4)) under RBD?*”

**Answer.** We agree that the contrast between the total normalization of SBP with Losartan alone and the diminished effect of this drug after a single administration of Ang-(3–4) deserves special consideration. For this reason, the data in Figure 7 has been presented in more detail in the RM, where pressoric values are explicitly mentioned (page 13, lines 365 to 369). These effects are discussed on page 17, lines 496 to 511 (RM), where three possibilities are considered.

**Comment 3.**

**Citation.** “*In figure 11-12, the ouabain-sensitive Na+ ATPase and the ouabain-resistant Na+ ATPase activities changes differently under RBD. What’s the different roles between the two kinds of Na+ ATPase? Please give more explanation in the Discussion.*”

**Answer.** The Discussion section in the RM compares the two kinds of Na+-transporting ATPases in proximal tubule cells and describes their corresponding roles (page 18, lines 535 to 540; page 18, lines 542 to 544). Four new references (# 56 to # 59) add support to the modified Discussion. These new references are highlighted in green in both, the text and list.
