# Supplementary material for: Angiotensin-(3–4) normalizes the elevated arterial blood pressure and abnormal Na+/energy handling associated with chronic undernutrition by counteracting the effects mediated by type 1 angiotensin II receptors
Source: PLoS One. 2022 Aug 19;17(8):e0273385. doi: 10.1371/journal.pone.0273385 (PMC9390919; doi:10.1371/journal.pone.0273385)
Supplement: Attachment — Submitted filename: Response to Reviewers PONE-D-22-09686 R1.docx [file pone.0273385.s002.docx]

**Response to Reviewers**

**Manuscript PONE-D-22-09686 R1**

The manuscript “*Angiotensin-(3–4) normalizes the elevated arterial blood pressure and abnormal Na^+^/energy handling associated with chronic undernutrition by counteracting the effects mediated by type 1 angiotensin II receptor”* has been carefully revised to meet the criticism raised by Reviewer 1.

**Reviewer 1.**

**Citation**. *"P> 0.05" is not appropriate because the authors declare that "significant difference was set to P <0.05". The authors should use P ≥ 0.05 instead of P > 0.05. If they simply want to express that there is no statistical difference, the reviewer recommends using NS (not significant). Otherwise, each figure will be confused. In other articles, the P value is expressed to the second decimal place even if there is no significant difference.*

**Answer.** We accept the recommendation by the Reviewer. The criticism has been addressed in two parts in the R2 version of the manuscript.

1) We replaced p > 0.05 by p *≥* 0.05 on page 10, line 286 (Word file) of the now uploaded R2 version. In the Statistic analysis section we define that “ns” is used when p ≥ 0.05 (page 9, line 242; R2 version).
2) Figures 2A, 2C, 3A, 3B, 3C, 4A, 4B, 4C, 5A, 5C, 7A, 7B, 7C, 8A, 8B, 8D, 9A, 9B, 9C, 9D, 10A, 10B, 10C, 10D, 11A, 11C, 12B, 12C, 12D, 13A, 13B, 13C, 14A, 14B and 14C were redrawn using “ns” between bars when no significant differences exist.

We thank again the careful review by the Academic Editor and the two Reviewers during the two rounds of analysis.
